# Supplementary material for: A Million-Cow Validation of a Chromosome 14 Region Interacting with All Chromosomes for Fat Percentage in U.S. Holstein Cows
Source: Int J Mol Sci. 2024 Jan 4;25(1):674. doi: 10.3390/ijms25010674 (PMC10779465; doi:10.3390/ijms25010674)
Supplement: Supplementary file 1 [file ijms-25-00674-s001.zip › Supplementary_material.pdf]

**Table S1.** Significant inter-chromosome A×A effects with log10(1/p)>32. (Excel file)

(AC1-AC4 are the four allelic combinations of the two loci. aa1-aa4 are the A×A epistasis values of AC1-AC4. frq1-frq4 are the frequencies of AC1-AC4.)

**Table S2.** Basic statistics of AFC phenotypic residuals after removing fixed nongenetic effects from the 12/2022 U.S. Holstein genetic evaluation.

| Trait | Number of cows | Mean (%) | Min (%) | Max (%) | SD (%) |
|---|---|---|---|---|---|
| FPC | 1,231,898 | -0.042 | -1.712 | 4.938 | 0.179 |

**Figure S1.** Circular plots of A×A effects between Chr14 and other chromosomes, and Manhattan plots of the statistical significance of A×A effects of all non-Chr14 chromosomes.

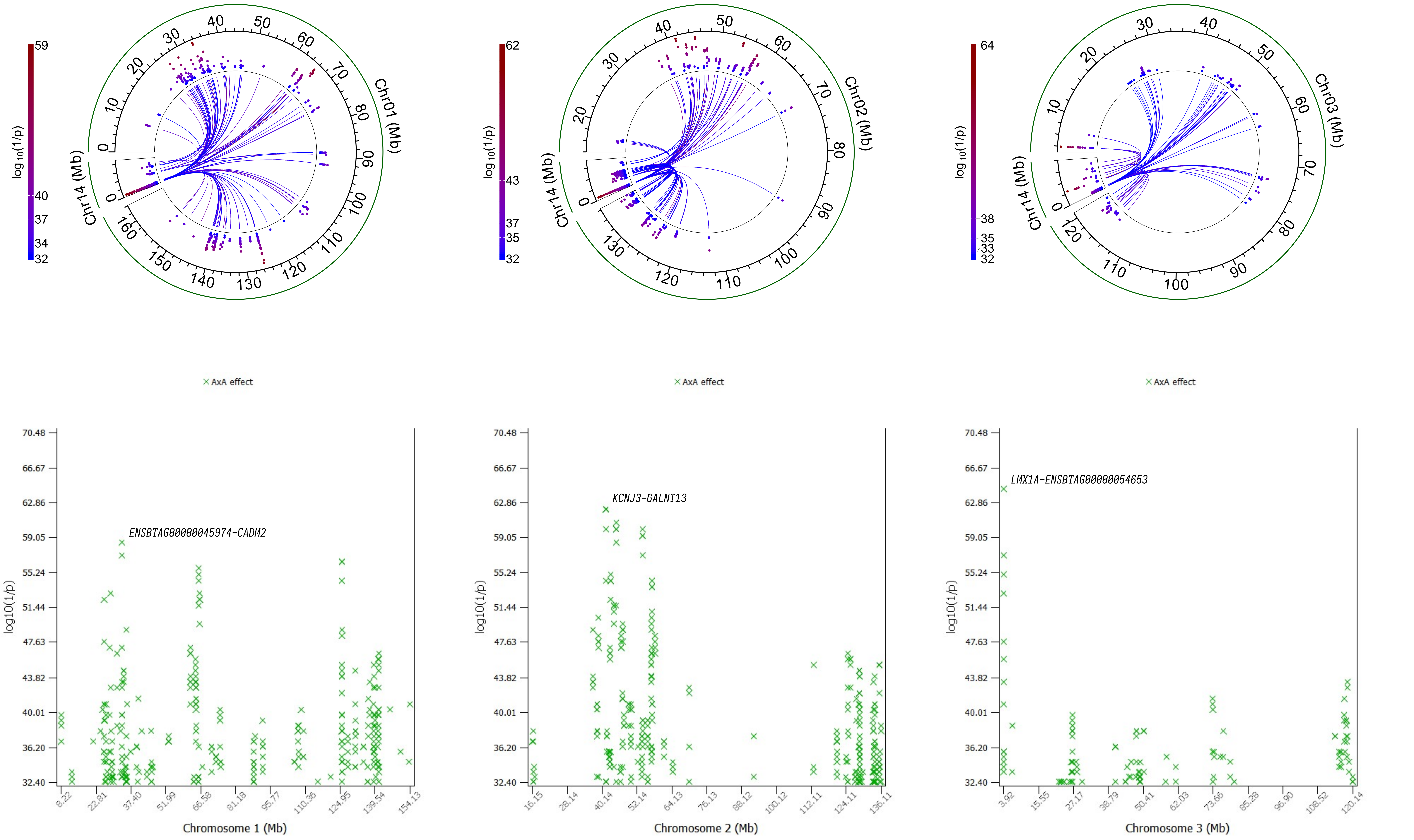

Chr01 (Mb)
Chr14 (Mb)
log10(1/p)
59
40
37
34
32
Chr02 (Mb)
62
43
37
35
32
Chr03 (Mb)
64
38
35
33
32
AxA effect
AxA effect
AxA effect
ENSBTAG00000045974-CADM2
KCNJ3-GALNT13
LMX1A-ENSBTAG00000054653
70.48
66.67
62.86
59.05
55.24
51.44
47.63
43.82
40.01
36.20
32.40
log10(1/p)
8.22
22.81
37.40
51.99
66.58
81.18
95.77
110.36
124.95
139.54
154.13
Chromosome 1 (Mb)
16.15
28.14
40.14
52.14
64.13
76.13
88.12
100.12
112.11
124.11
136.11
Chromosome 2 (Mb)
3.92
15.55
27.17
38.79
50.41
62.03
73.66
85.28
96.90
108.52
120.14
Chromosome 3 (Mb)

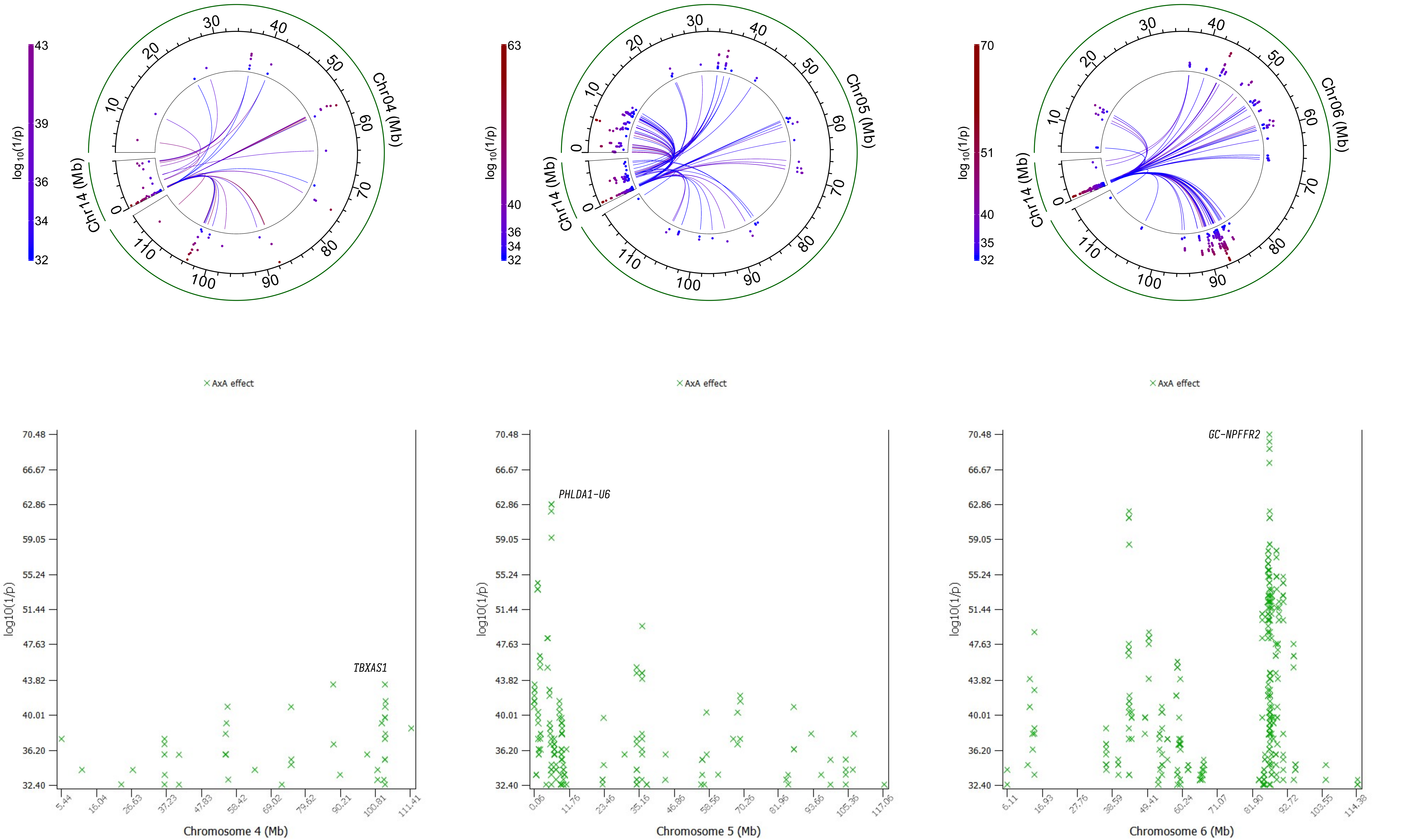

Chr04 (Mb)
Chr05 (Mb)
Chr06 (Mb)
Chr14 (Mb)
log10(1/p)
AxA effect
TBXAS1
PHLDA1-U6
GC-NPFFR2
Chromosome 4 (Mb)
Chromosome 5 (Mb)
Chromosome 6 (Mb)

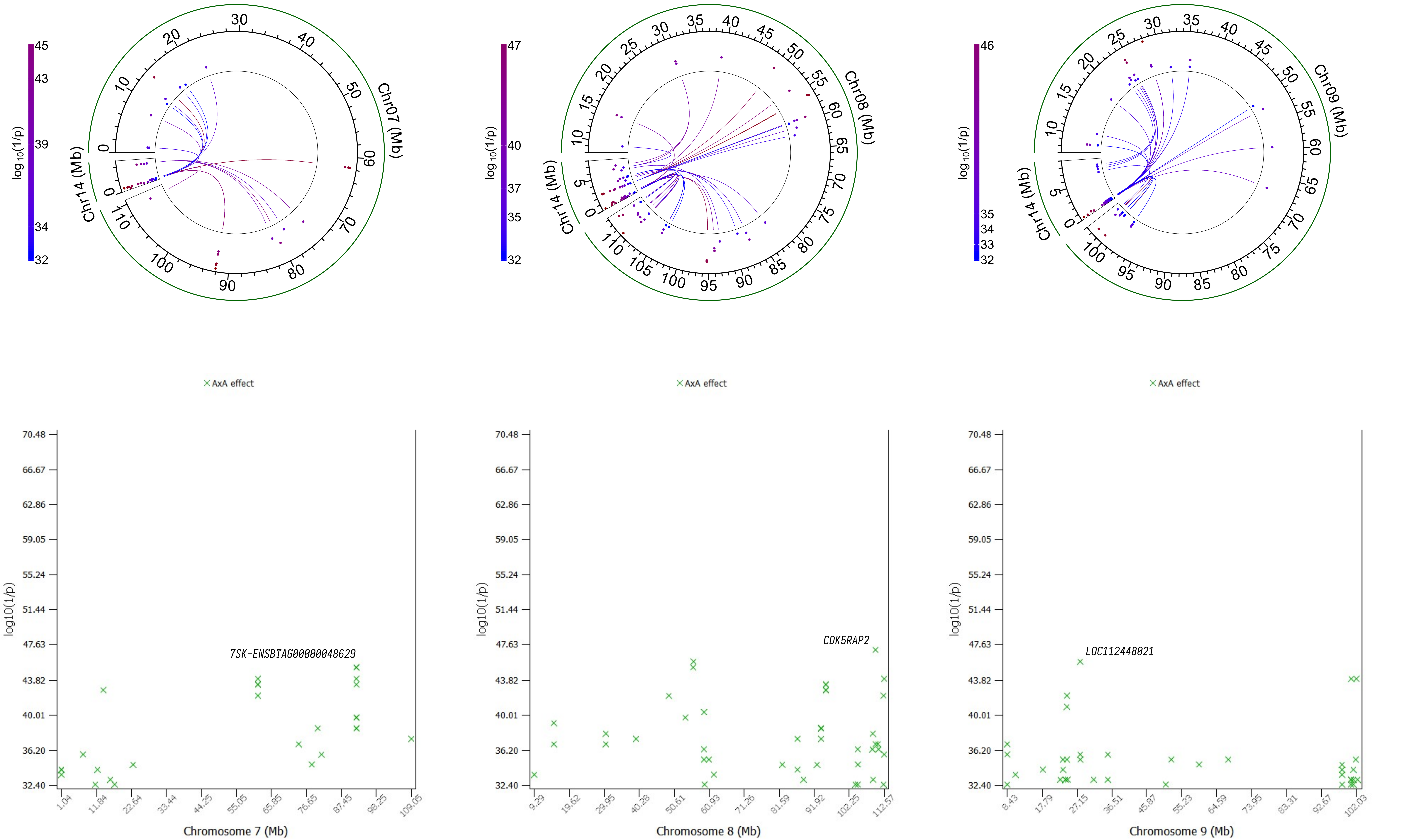

log10(1/p)
Chr14 (Mb)
Chr07 (Mb)
Chr08 (Mb)
Chr09 (Mb)
AxA effect
7SK-ENSBTAG00000048629
CDK5RAP2
LOC112448021
Chromosome 7 (Mb)
Chromosome 8 (Mb)
Chromosome 9 (Mb)

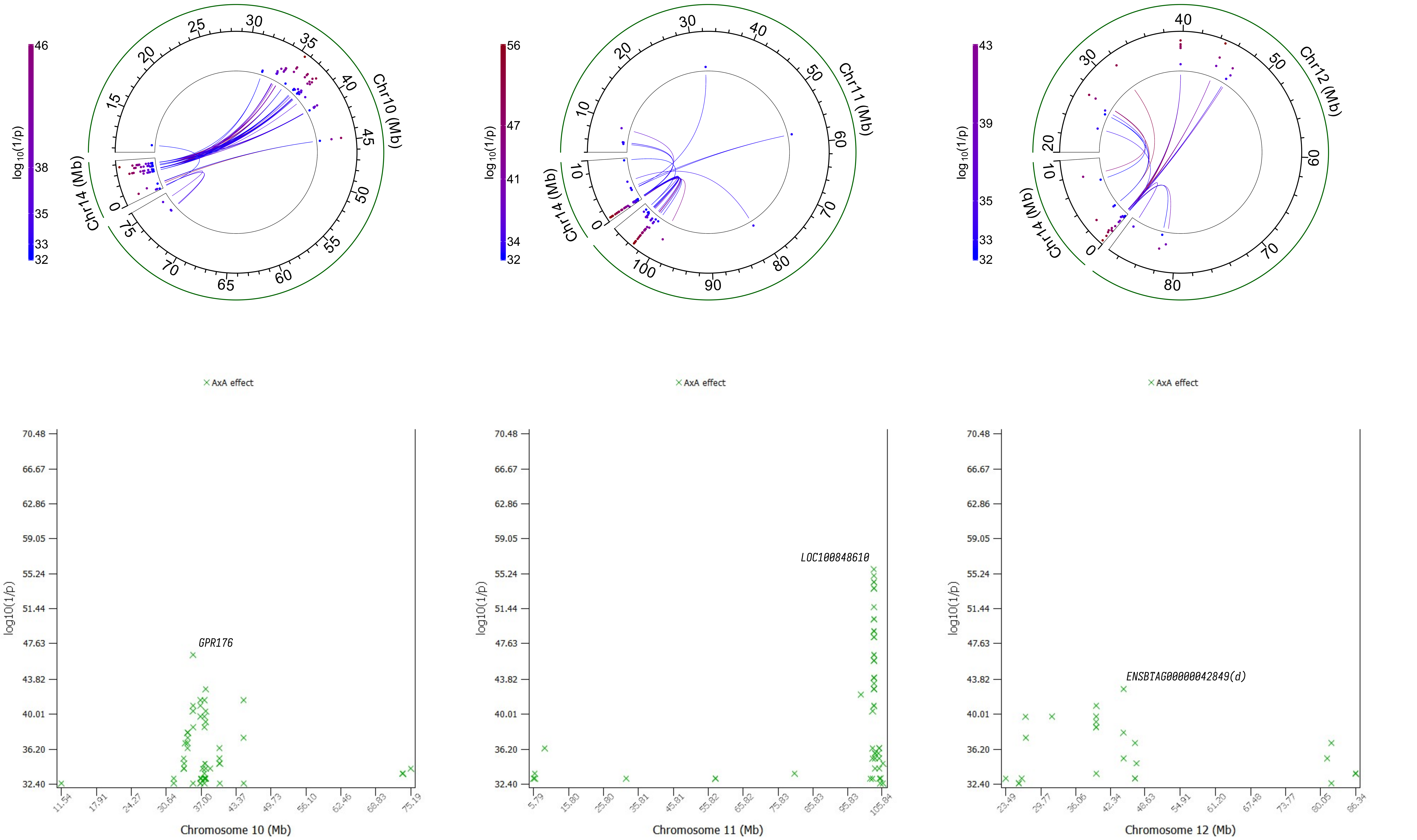

log10(1/p)
46
38
35
33
32
Chr10 (Mb)
Chr14 (Mb)
56
47
41
34
32
Chr11 (Mb)
Chr14 (Mb)
43
39
35
33
32
Chr12 (Mb)
Chr14 (Mb)
AxA effect
AxA effect
AxA effect
70.48
66.67
62.86
59.05
55.24
51.44
47.63
43.82
40.01
36.20
32.40
log10(1/p)
GPR176
11.54
17.91
24.27
30.64
37.00
43.37
49.73
56.10
62.46
68.83
75.19
Chromosome 10 (Mb)
LOC100848610
5.79
15.80
25.80
35.81
45.81
55.82
65.82
75.83
85.83
95.83
105.84
Chromosome 11 (Mb)
ENSBTAG00000042849(d)
23.49
29.77
36.06
42.34
48.63
54.91
61.20
67.48
73.77
80.05
86.34
Chromosome 12 (Mb)

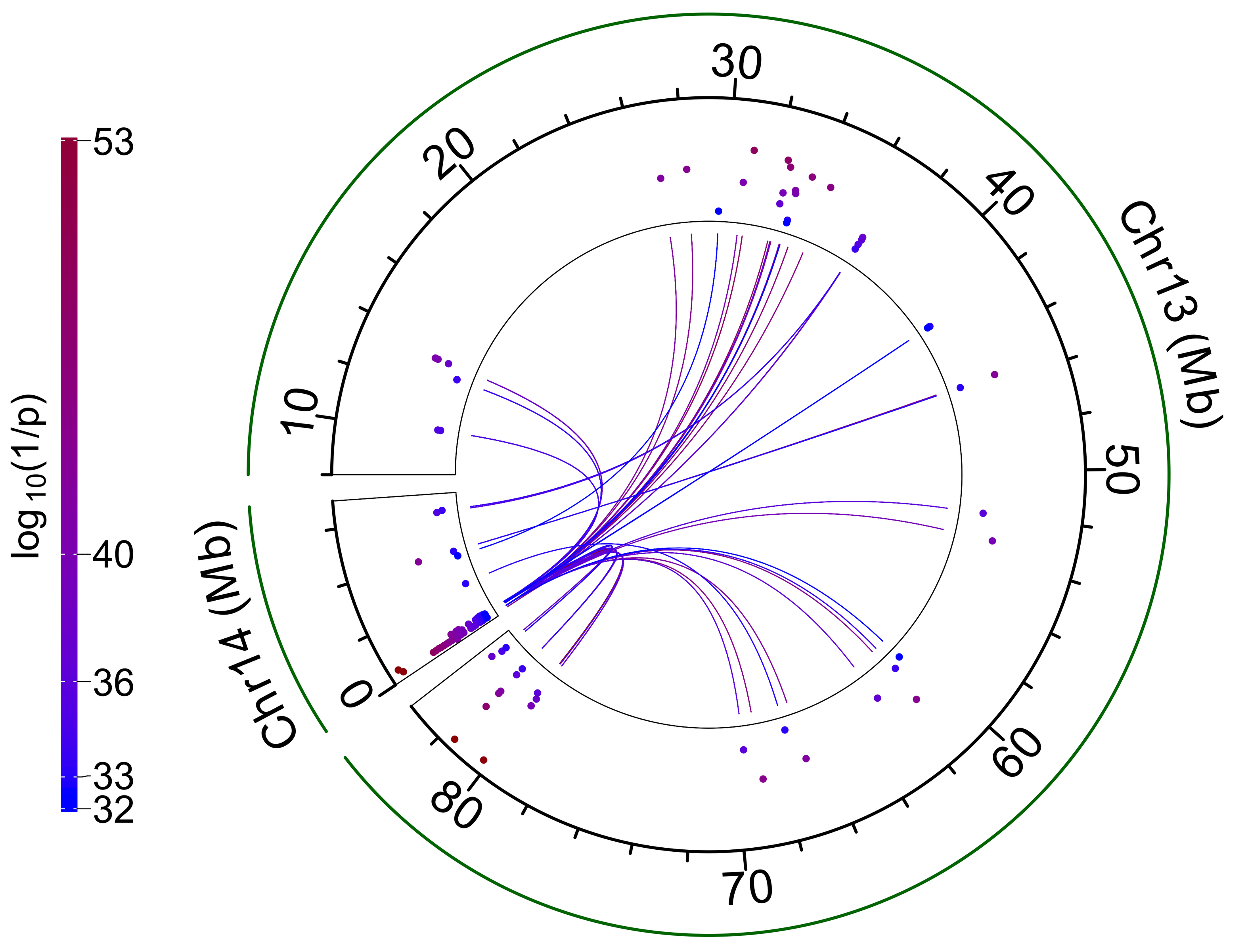
log10(1/p)
53
40
36
33
32
Chr13 (Mb)
Chr14 (Mb)
0
10
20
30
40
50
60
70
80

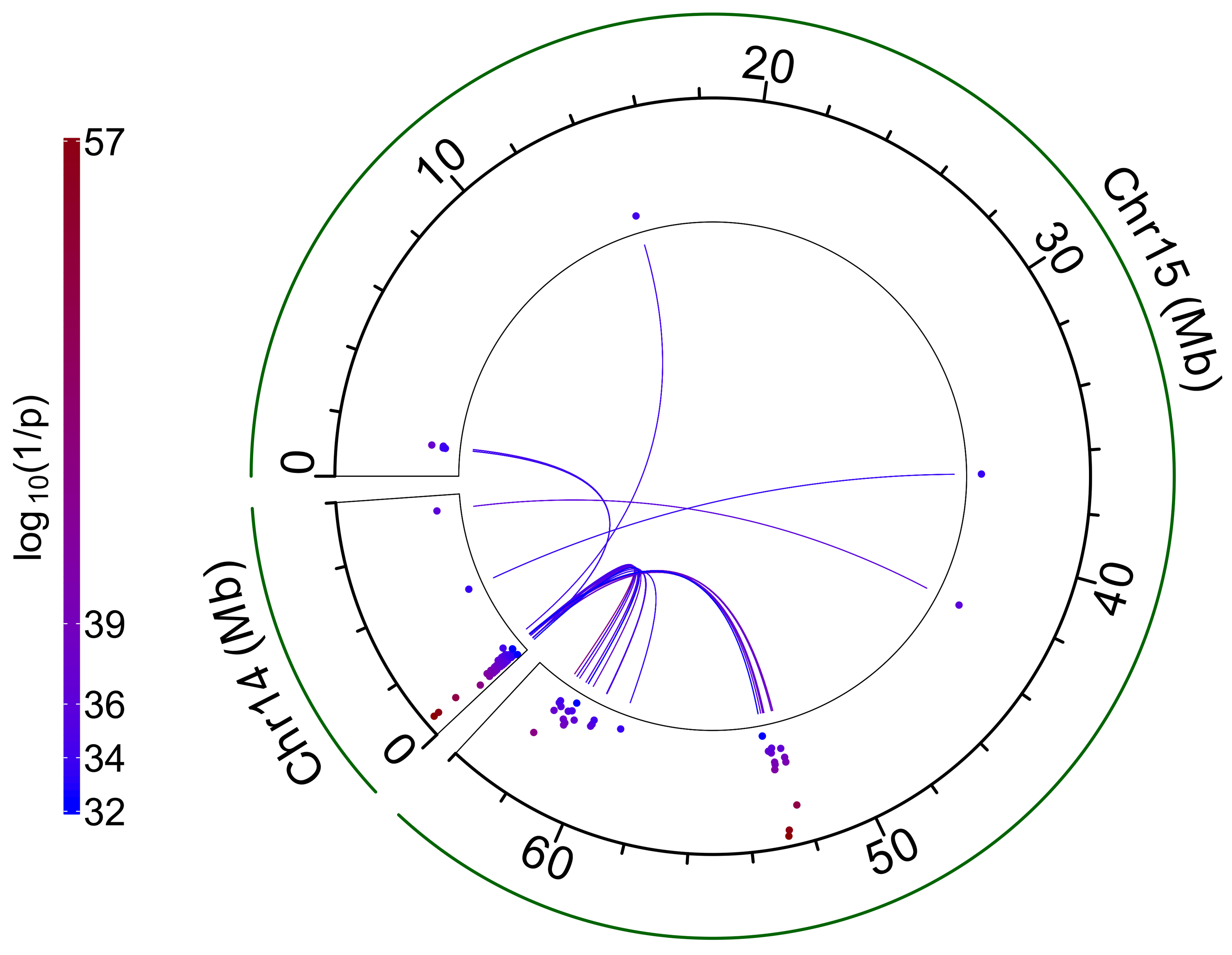
log10(1/p)
57
39
36
34
32
Chr15 (Mb)
Chr14 (Mb)
0
10
20
30
40
50
60

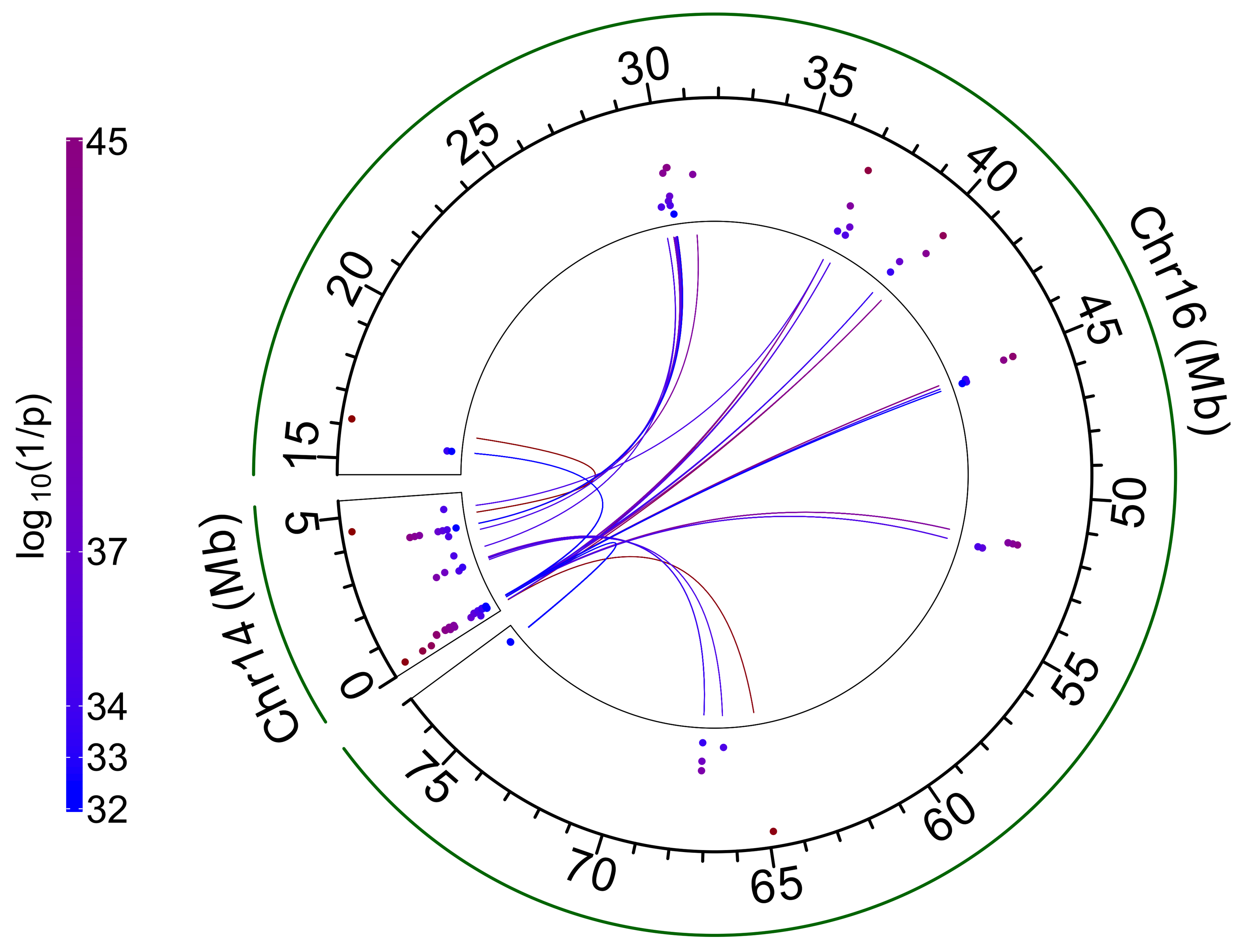
log10(1/p)
45
37
34
33
32
Chr16 (Mb)
Chr14 (Mb)
0
5
15
20
25
30
35
40
45
50
55
60
65
70
75

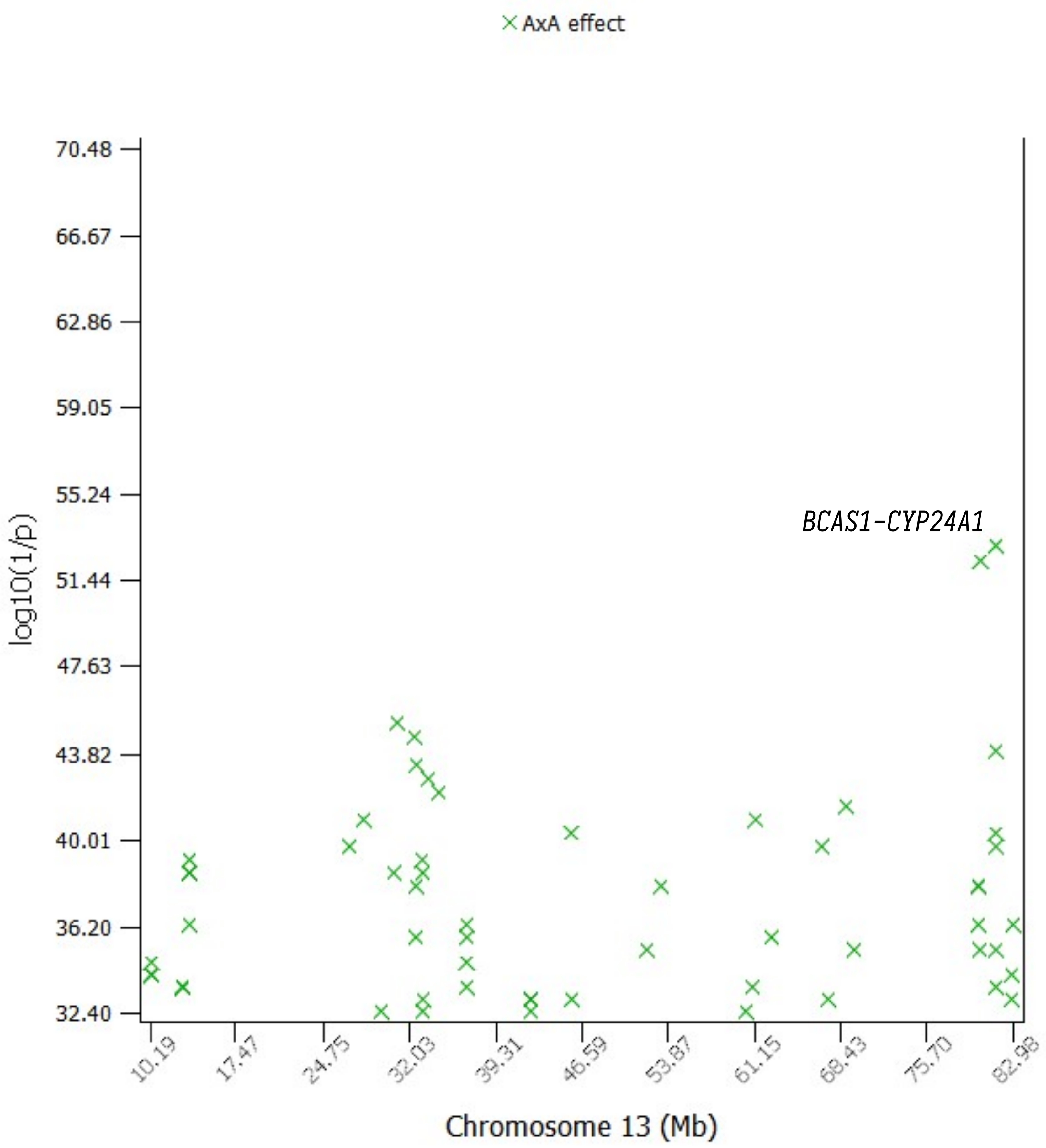
AxA effect
log10(1/p)
70.48
66.67
62.86
59.05
55.24
51.44
47.63
43.82
40.01
36.20
32.40
BCAS1-CYP24A1
10.19
17.47
24.75
32.03
39.31
46.59
53.87
61.15
68.43
75.70
82.98
Chromosome 13 (Mb)

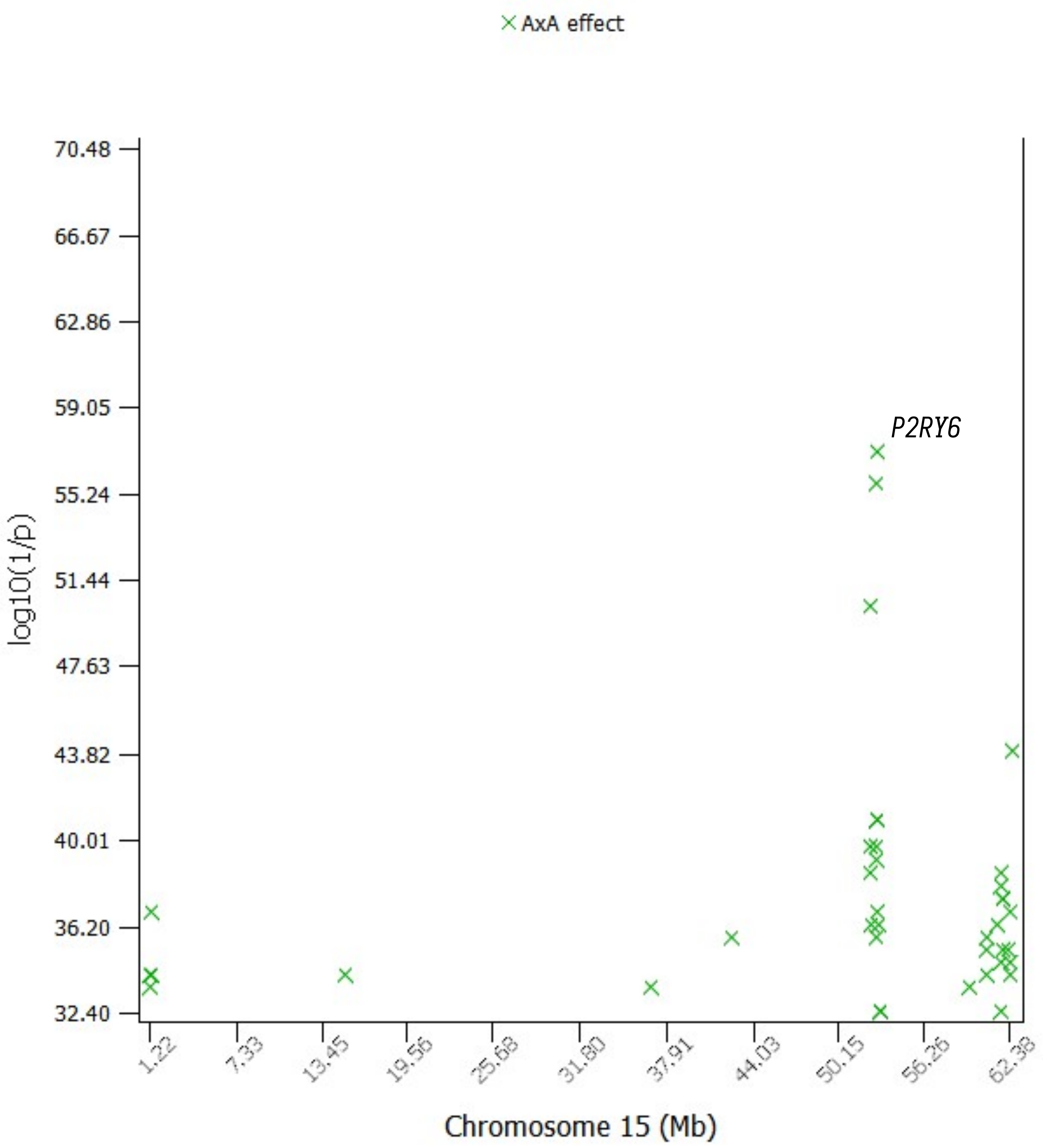
AxA effect
log10(1/p)
70.48
66.67
62.86
59.05
55.24
51.44
47.63
43.82
40.01
36.20
32.40
P2RY6
1.22
7.33
13.45
19.56
25.68
31.80
37.91
44.03
50.15
56.26
62.38
Chromosome 15 (Mb)

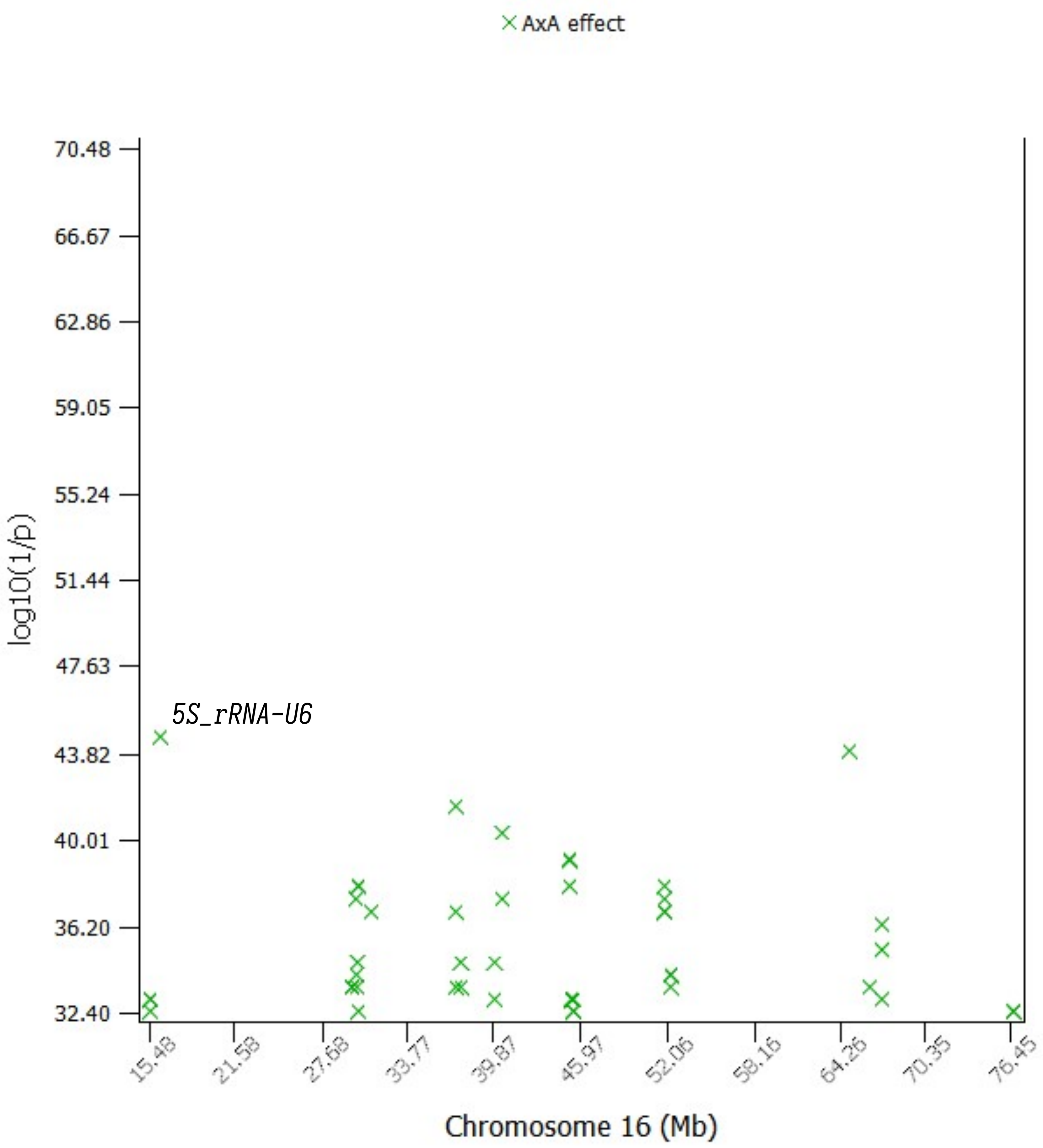
AxA effect
log10(1/p)
70.48
66.67
62.86
59.05
55.24
51.44
47.63
43.82
40.01
36.20
32.40
5S_rRNA-U6
15.48
21.58
27.68
33.77
39.87
45.97
52.06
58.16
64.26
70.35
76.45
Chromosome 16 (Mb)

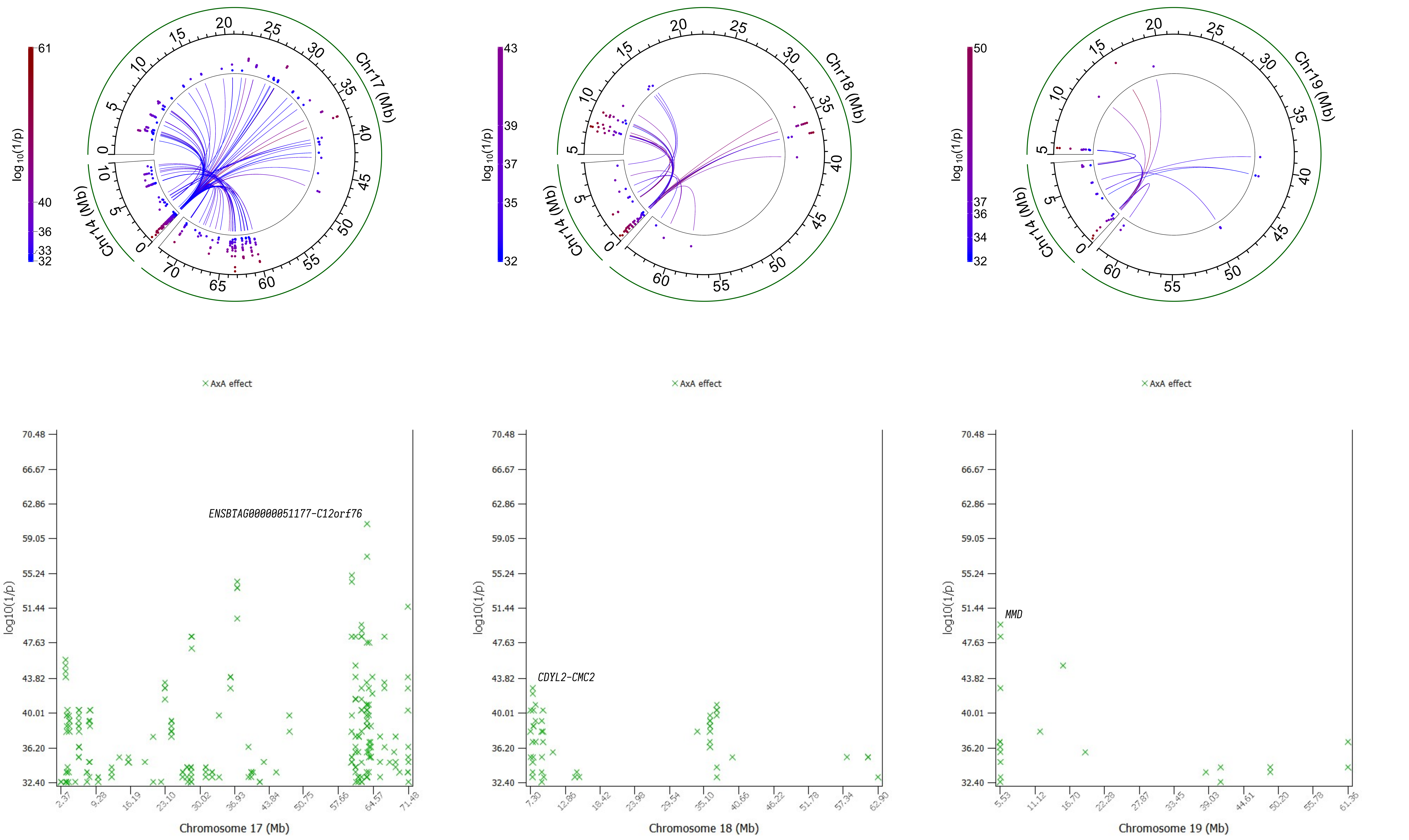
log10(1/p)
Chr17 (Mb)
Chr14 (Mb)
Chr18 (Mb)
Chr14 (Mb)
Chr19 (Mb)
Chr14 (Mb)
AxA effect
AxA effect
AxA effect
ENSBTAG00000051177-C12orf76
CDYL2-CMC2
MMD
log10(1/p)
Chromosome 17 (Mb)
Chromosome 18 (Mb)
Chromosome 19 (Mb)

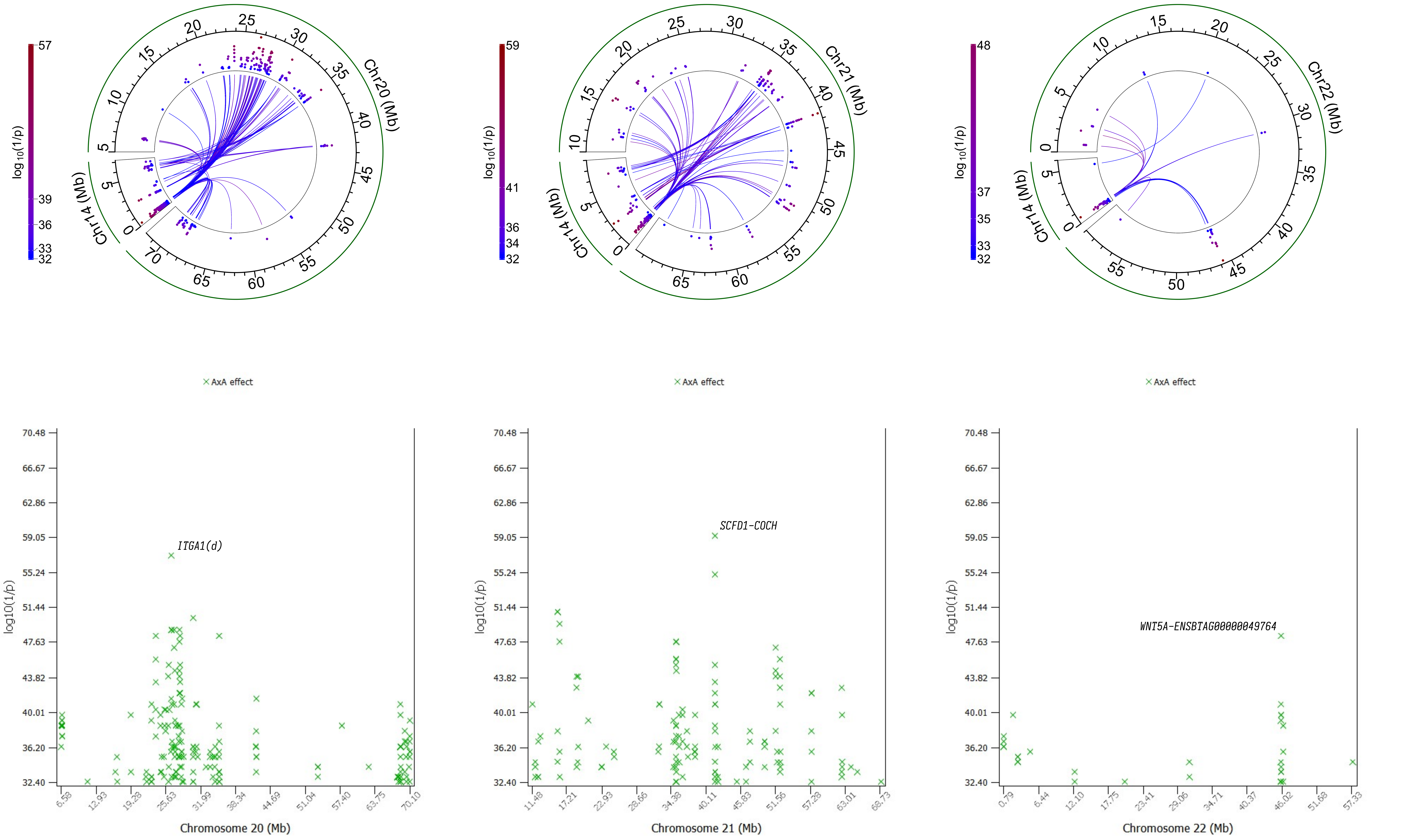

Chr20 (Mb)
Chr14 (Mb)
log10(1/p)
Chr21 (Mb)
Chr22 (Mb)
AxA effect
ITGA1(d)
SCFD1-COCH
WNT5A-ENSBTAG00000049764
Chromosome 20 (Mb)
Chromosome 21 (Mb)
Chromosome 22 (Mb)

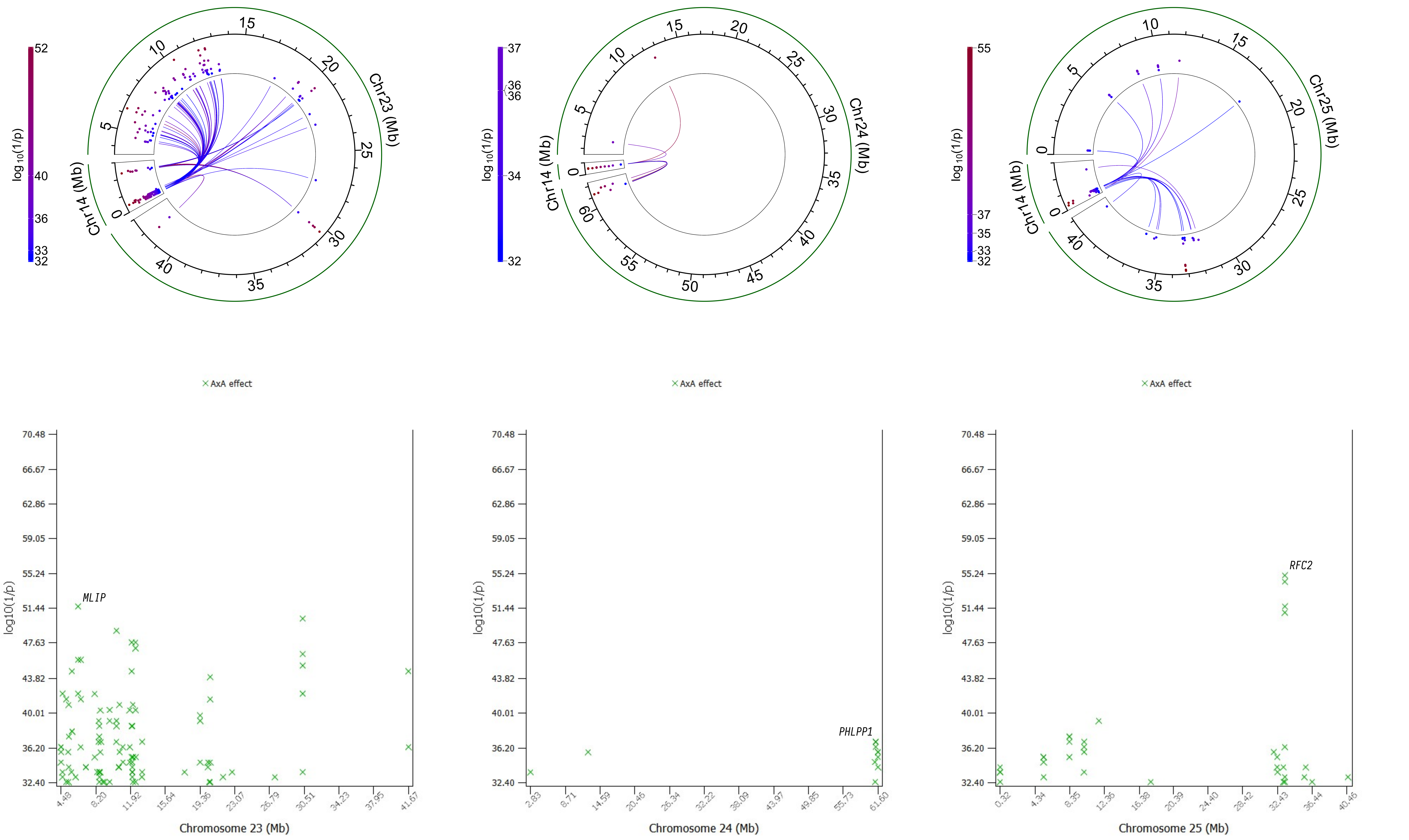

log10(1/p)
Chr14 (Mb)
Chr23 (Mb)
Chr24 (Mb)
Chr25 (Mb)
AxA effect
MLIP
PHLPP1
RFC2
Chromosome 23 (Mb)
Chromosome 24 (Mb)
Chromosome 25 (Mb)

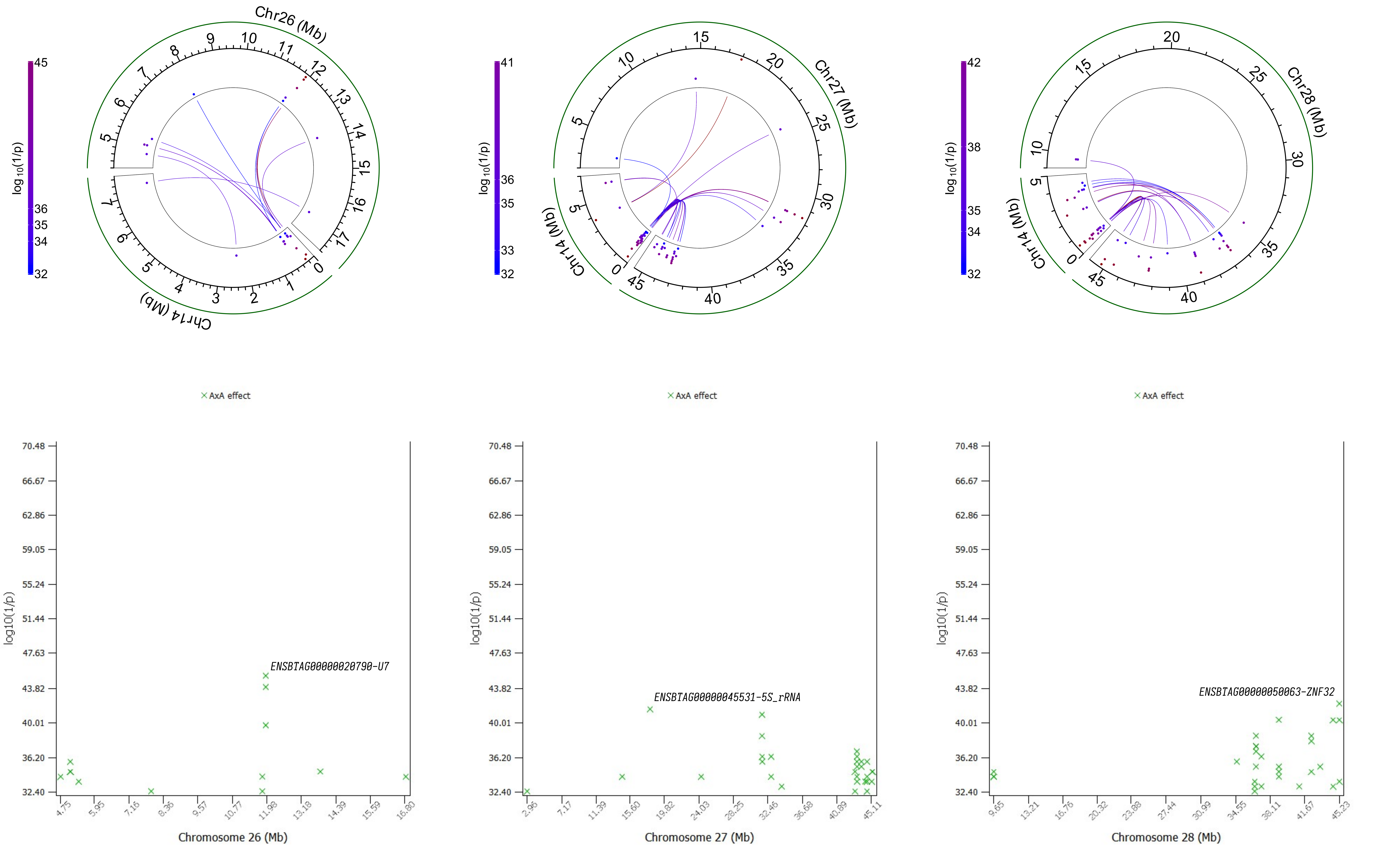

Chr26 (Mb)
Chr14 (Mb)
log10(1/p)
45
36
35
34
32
Chr27 (Mb)
41
33
Chr28 (Mb)
42
38
AxA effect
ENSBTAG00000020790-U7
ENSBTAG00000045531-5S_rRNA
ENSBTAG00000050063-ZNF32
log10(1/p)
70.48
66.67
62.86
59.05
55.24
51.44
47.63
43.82
40.01
36.20
32.40
Chromosome 26 (Mb)
4.75
5.95
7.16
8.36
9.57
10.77
11.98
13.18
14.39
15.59
16.80
Chromosome 27 (Mb)
2.96
7.17
11.39
15.60
19.82
24.03
28.25
32.46
36.68
40.89
45.11
Chromosome 28 (Mb)
9.65
13.21
16.76
20.32
23.88
27.44
30.99
34.55
38.11
41.67
45.23

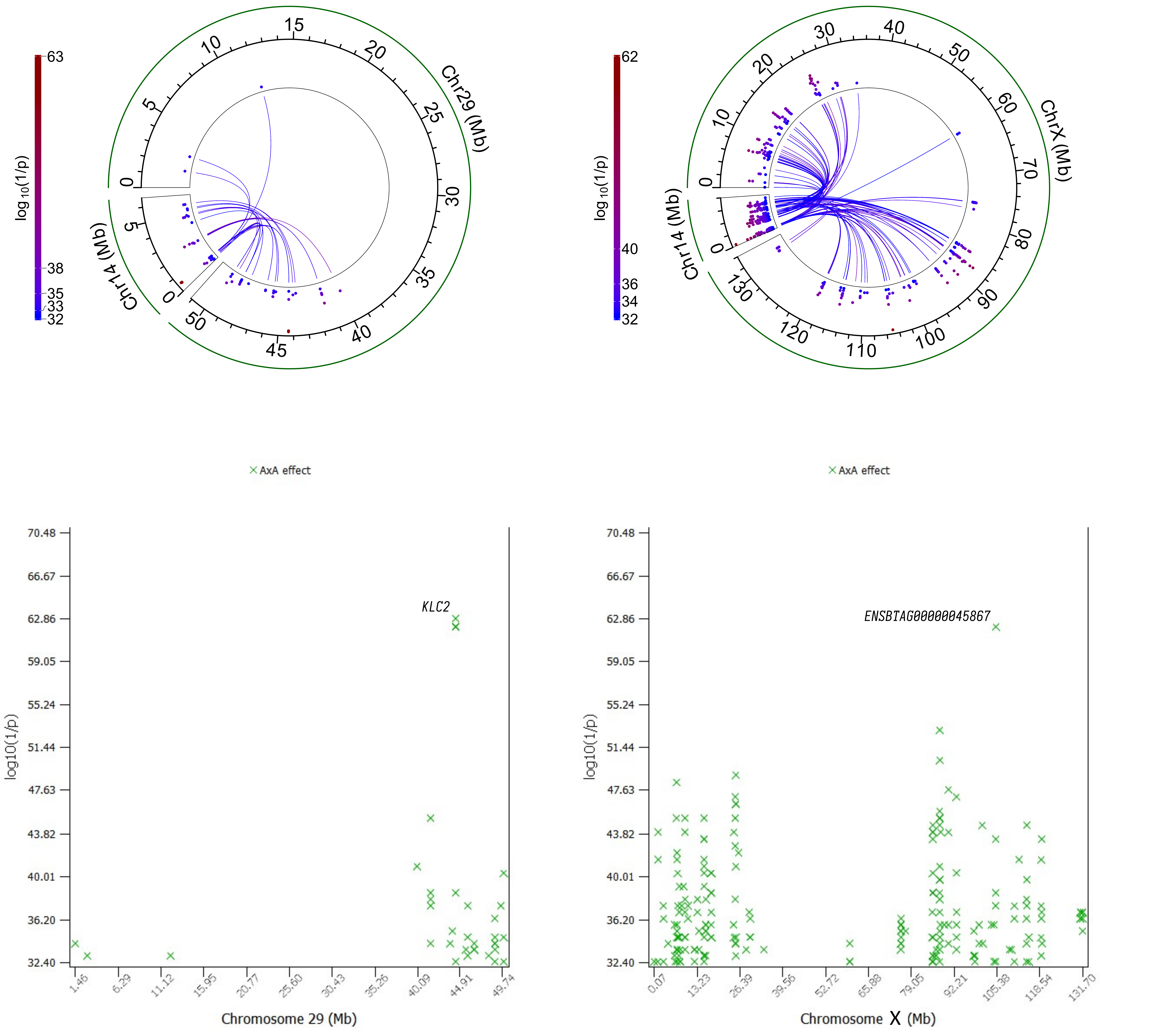

Chr29 (Mb)
Chr14 (Mb)
log10(1/p)
63
38
35
33
32
ChrX (Mb)
62
40
36
34
AxA effect
KLC2
ENSBTAG00000045867
70.48
66.67
62.86
59.05
55.24
51.44
47.63
43.82
40.01
36.20
32.40
1.46
6.29
11.12
15.95
20.77
25.60
30.43
35.26
40.09
44.91
49.74
Chromosome 29 (Mb)
0.07
13.23
26.39
39.56
52.72
65.88
79.05
92.21
105.38
118.54
131.70
Chromosome X (Mb)
